# Cortactin deacetylation by HDAC6 and SIRT2 regulates neuronal migration and dendrite morphogenesis during cerebral cortex development

**DOI:** 10.1186/s13041-020-00644-y

**Published:** 2020-07-25

**Authors:** Ji-ye Kim, Hee-Gon Hwang, Joo-Yong Lee, Minkyu Kim, Jeong-Yoon Kim

**Affiliations:** 1 Department of Microbiology and Molecular Biology, Chungnam National University, Daejeon, South Korea; 2 Graduate School of Analytical Science and Technology (GRAST), Chungnam National University, Daejeon, South Korea; 3 Divison of Animal and Dairy Science, Chungnam National University, Daejeon, South Korea

**Keywords:** Dendrite development, Neuronal migration, HDAC6, Cortactin deacetylation, The Golgi apparatus

## Abstract

Proper dendrite morphogenesis and neuronal migration are crucial for cerebral cortex development and neural circuit formation. In this study, we sought to determine if the histone deacetylase HDAC6 plays a role in dendrite development and neuronal migration of pyramidal neurons during cerebral cortex development. It was observed that knockdown of HDAC6 leads to defective dendrite morphogenesis and abnormal Golgi polarization in vitro, and the expression of wild type cortactin or deacetyl-mimetic cortactin 9KR rescued the defective phenotypes of the HDAC6 knockdown neurons. This suggests that HDAC6 promotes dendritic growth and Golgi polarization through cortactin deacetylation in vitro. We also demonstrated that ectopic expression of SIRT2, a cytoplasmic NAD^+^ − dependent deacetylase, suppresses the defects of HDAC6 knockdown neurons. These results indicate that HDAC6 and SIRT2 may be functionally redundant during dendrite development. Neurons transfected with both HDAC6 and SIRT2 shRNA or acetyl-mimetic cortactin 9KQ showed slow radial migration compared to the control cells during cerebral cortex development. Furthermore, a large portion of cortactin 9KQ-expressing pyramidal neurons at layer II/III in the cerebral cortex failed to form an apical dendrite toward the pial surface and had an increased number of primary dendrites, and the percentage of neurons with dendritic Golgi decreased in cortactin 9KQ-expressing cells, compared to control neurons. Taken together, this study suggests that HDAC6 and SIRT2 regulate neuronal migration and dendrite development through cortactin deacetylation in vivo.

## Introduction

Neurons are the most highly polarized cells in our body. They dynamically change shape throughout the process of neuron differentiation, neuronal cell migration, and the formation of functional networks during cerebral cortex development. Neurons have morphologically distinct axon and dendrites, which are optimized for their specific functional roles. The complex morphologies underlie their ability to integrate synaptic inputs and outputs [1, 2]. Additionally, the morphological change from the multipolar to the bipolar shape of newly born neurons in the cortex is critical for normal neuronal migration during cerebral cortex development [3, 4]. For these reasons, the mechanisms that regulate neuronal morphogenesis and migration during cortex development have been investigated extensively over recent decades [5, 6].

At the onset of neurogenesis during cerebral cortex development, neural stem cells transform into radial glial cells (RGCs). RGCs symmetrically divide to generate self-renewing RGCs and begin to asymmetrically divide to produce young neurons or intermediate progenitors that are the source of newborn neurons [7–9]. Newly generated neurons in the subventricular and intermediate zones undergo dramatic morphological changes. These changes are important for proper neuronal migration to reach near the top of the cortical plate. During asymmetric cell division, newly generated neurons begin to have a multipolar morphology and initially randomly migrate radially, but ultimately migrate toward the cortical plate [10, 11]. By the time cell bodies reach the cortical plate, the neurons have adopted a bipolar morphology with an axon and a leading process. The neurons then attach to and migrate along the radial glial fiber toward the pial surface, through a process called radial neuronal migration [3]. If the multipolar-to-bipolar transition does not occur properly, the radial migration is delayed and eventually results in aberrant neural connectivity and psychiatric disorders, such as schizophrenia and autism spectrum disorders [12, 13]. Therefore, accurately identifying factors that influence neuronal cell morphology during cerebral cortex development is necessary to gain a better understanding of the possible molecular mechanisms underlying neurogenesis in the brain.

Histone deacetylase 6 (HDAC6) is one of the HDAC family proteins that can be subdivided into the following four classes: class I (HDAC1, 2, 3, and 8), class II (IIa: HDAC4, 5, 7, and 9; IIb: HDAC6 and 10), class III (sirtuin 1–7) and class IV (HDAC11) [14]. HDAC6 is mainly localized in the cytoplasm in order to deacetylate non-histone target proteins, such as α-tubulin, cortactin, and HSP90. Consequently, HDAC6 regulates various critical cellular functions, including cilia formation, cell motility, metastasis, and autophagosome maturation [15–19]. Especially, HDAC6 deacetylates the α-tubulin K40 residue to make dynamic microtubules [17] and the lysine residues in the repeat region of cortactin to recruit the actin-nucleating complex Arp2/3 for actin branching and polymerization [19–21]. Despite the importance of the functions of α-tubulin, cortactin, and HSP90 during development, HDAC6-deficient mice are viable with normal development and show no noticeable defects in brain morphology, motor coordination, or hippocampal dependent cognitive functions [22, 23]. However, it has been previously reported that HDAC6 has important roles in both neuroprotection and neurodegeneration [24]. Expression of HDAC6 has been reported to rescue neurodegeneration associated with the ubiquitin-proteasome system dysfunction and promote alpha-synuclein inclusion in a Parkinson’s disease model [25, 26]. Conversely, HDAC6 inhibition compensates for the transport deficit found in Huntington’s disease; reverses the axonal loss in a Charcot-Marie-Tooth disease model mouse; and suppresses neuritic tau pathology and cognitive deficits in Alzheimer’s disease models [23, 27–31]. These seemingly contradictory roles may be due to the HDAC6’s deacetylation or ubiquitin-dependent activities that regulate a wide range of cellular processes.

Additionally, HDAC6 appears to influence neuronal cell morphogenesis. It has been reported that HDAC6 plays role in the differentiation of dendrites by stimulating the activity of the centrosomal Cdc20-APC complex [32] and is required for dendritic maturation of newly generating neurons after ischemic stroke [33]. Furthermore, it is has been reported that HDAC6 deacetylates α-tubulin and cortactin, which regulate cytoskeletal dynamics for the functional morphology of neurons [34, 35]. Therefore, we sought to determine if HDAC6 plays a role in neuronal cell morphogenesis during cerebral cortex development. In this study, we demonstrate that HDAC6 regulates dendrite development and dendritic Golgi polarization through deacetylation of cortactin in cultured hippocampal neurons. Furthermore, we demonstrate that cortactin deacetylation by HDAC6, together with SIRT2, is required for radial migration and dendrite development in the cerebral cortex.

## Materials and methods

### Plasmids

Tubulin K40Q, tubulin K40R expression vectors were gifts from Kenneth Yamada (Addgene plasmid #105302, #105303). SIRT2 shRNA was designed against mouse and rat SIRT2 mRNA targeting the coding sequence (CDS). The sequence of the target was: 5′-GGAGCATGCCAACATAGATGC-3′. Oligonucleotides were annealed and ligated into the pSUPER vector (Oligoengine) and a modified pSUPER-Venus vector that expresses Venus (YFP) under control of the CMV promoter. For expressing SIRT2 resistant to shRNA, full-length human SIRT2 was amplified by PCR and subcloned into expression vector. HDAC6 mutant resistant to shRNA was generated by PCR with primer mutating the HDAC6 shRNA target site. To generate shRNA resistant HDAC6, the HDAC6 shRNA target sequence, 5′-**T**CT**A**GC**G**GAGGTAAAGAAG-3′, was replaced with **C**CT**G**GC**C**GAGGTAAAGAAG-3′ that carries silent mutations. All constructs were verified by DNA sequencing. For in vivo expression via in utero electroporation, tdTomato, Venus tagged GalT2 and cortactin 9KQ were subcloned into the pCAGGS vector containing a chicken-β-actin (CAG) promoter.

### Antibodies

Primary antibodies used were as follows: anti-GM130 (1:4000, BD Biosciences), anti-AnkG (1:2000, Abcam), anti-HDAC6 (1:1000, Santa Cruz Biotechnology and Cell Signaling Technology), anti-cortactin (1:3000, Merck), anti-acetyl cortactin (1:1000, Merck), anti-Goat anti-mouse IgG Alexa Fluor 594-labeled secondary antibody (1:4000, Thermo Fisher Scientific), and Goat anti-mouse IgG Alexa Fluor 488-labeled secondary antibody (1:4000, Thermo Fisher Scientific).

### Primary neuron culture

All animal protocols were reviewed and approved by the Institutional Animal Care and Use Committee of Chungnam National University. Primary neurons were cultured according to previously described protocols [36]. Briefly, hippocampi were dissected from rat embryos (embryonic [E] day 19), trypsinized, and triturated through a glass Pasteur pipette. The dissociated neurons were plated at a concentration of 150,000 cells per 60-mm dish containing four 18-mm glass coverslips (Thermo Fisher Scientific) previously coated with 1 mg/ml poly-L-lysine (Sigma). After initial attachment, the coverslips were transferred to dishes containing a monolayer of glial cells. Cells were maintained in neurobasal medium (Invitrogen) supplemented with B27 (Invitrogen) and 1× GlutaMAX (Invitrogen). Neurons were transfected by using electroporation (NEPA21) according to the manufacture’s recommendation. Briefly, dissociated neurons were resuspended in MEM medium (Invitrogen) with 10 μg of Endotoxin-free high-quality DNA. The mixture received two types of pulse, poring pulse and transfer pulse. The poring pulse condition was as follows: 275 V, 0.5 ms pulse length, total two pulses, 50 ms interval between the pulses, 10% decay. Rate with (+) polarity. The transfer pulse condition was as follows: 20 V, 50 ms pulse length, total five pulses, 50 ms interval among the pulses, 40% decay. Rate with (+/−) polarity. Neurons were fixed after 14 days in vitro (DIV) with 4% paraformaldehyde in PBS for 15 min at room temperature.

### In utero electroporation

Timed pregnant ICR mice were used for in utero electroporation as previously described with slight modification [37]. Briefly, the day of vaginal plug appearance was considered E 0.5. At E 14.5, dams were anesthetized with an intramuscular injection of zoletil/xylazine mixture. A midline incision was made, and the uterus was exposed. We injected 1–2 μl of plasmid DNA (final concentration of 2 μg/μl) mixed with 0.1% fast green dye (Carl Roth), intracerebrally using a pulled glass micropipette (outer diameter 35–45 μm). After injection, individual embryos were held by forceps-type electrodes (NEPA) targeting the cerebral cortex. The cortex received two types of pulse, as mentioned above. The poring pulse condition was as follows: 33 V, 50 ms pulse length, total four pulses, 950 ms interval among the pulses, 10% decay. Rate with (+) polarity. The transfer pulse condition was as follows: 10 V, 50 ms pulse length, total three pulses, 50 ms interval among the pulses, 40% decay. Rate with (+/−) polarity. After the procedure, the uterus was put back into the abdominal cavity, and gently sprayed with pre-warmed PBS. The abdominal wall and skin were sutured to allow the embryos to develop full-term. The entire surgical procedure was completed within 30 min. The following DNA solutions were used: 4 μg/μl pCAGGS-tdTomato, 4 μg/μl pSUPER, 4 μg/μl pSUPER-HDAC6 shRNA, 4 μg/μl pSUPER-SIRT2 shRNA, 4 μg/μl pCAG-cortactin 9KQ, and 4 μg/μl pCAG-venus-GalT2 vector.

### Immunohistochemistry

Cultured neurons on coverslips were fixed with 4% paraformaldehyde in PBS for 15 min at room temperature, blocked for 1 h in blocking solution (Roche) with 0.3% TritonX-100 in PBS, and incubated in diluted primary antibody solution overnight at 4 °C. Cells were washed the next day in PBS and incubated in diluted secondary antibodies for 2 h before the final washing. Coverslips were mounted with antifade reagent (Thermo Fisher Scientific).

Embryonic brains and perfused mouse post-natal (14 days) brains were fixed with 4% paraformaldehyde in PBS overnight at 4 °C, and then transferred to 20% sucrose in PBS overnight and changed to 30% sucrose in PBS overnight at 4 °C. Brains were embedded in O.C.T compound (Sakura). Brains were cryosectioned into 50 μm thick slices. Brain slices were washed 3 times in PBS and incubated in diluted Hoechst (Thermo Fisher Scientific) solution for 2 h before the final washing. After nuclei staining, brain slices were mounted with antifade reagent (Thermo Fisher Scientific).

### Quantification of dendrite complexity

Dendrite complexity was quantified using Sholl analysis that measures the number of intersections by drawing concentric circles every 1 μm based on the center of soma. Data were analyzed by ImageJ plugin Neuron J.

### Image acquisition

Images were captured using an Olympus BX51 microscope (DAPI excitation and emission, 330–385 nm and 420 nm; GFP excitation and emission, 460–490 nm and 520 nm; RFP excitation and emission 530–550 nm and 575 nm). In vivo data were captured using a Carl Zeiss LSM 880 with a Airyscan confocal microscope (DAPI excitation and emission, 405 nm and 444 nm; GFP excitation and emission, 488 nm and 522 nm; RFP excitation and emission, 561 nm, 604 nm). Images were modified using Image J.

### Statistics

All data are presented as mean ± S.E.M. Pairwise comparisons were analyzed using a two-tailed Student’s t-test. *P* values ≤0.05 were considered statistically significant.

## Results

### HDAC6 regulates dendrite development

**Fig. 1:**
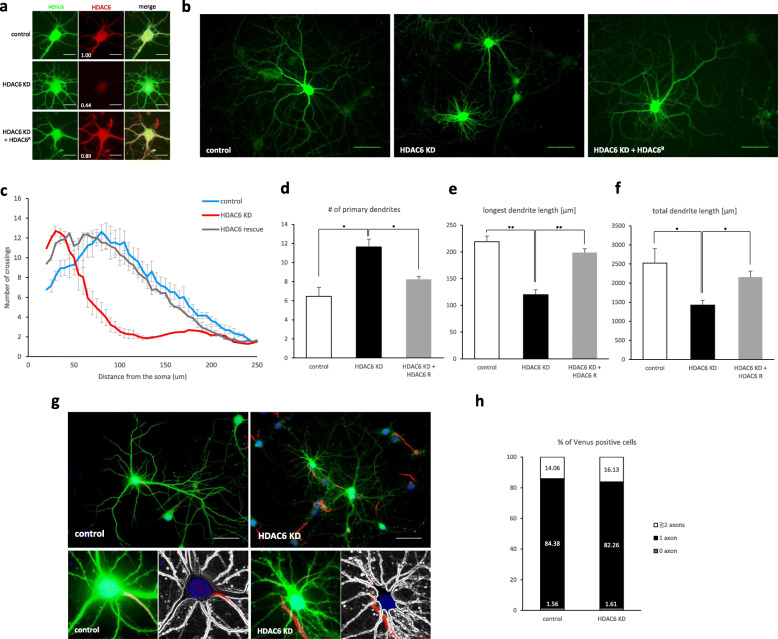
HDAC6 is required for dendrite development in hippocampal neurons. **a** Knockdown effect of HDAC6 shRNA on HDAC6 expression. The HDAC6 level (red) was decreased in HDAC6 shRNA expressing neurons (venus), but expression of HDAC6 shRNA resistant HDAC6 mutant recovered the HDAC6 level in HDAC6 knockdown neurons. Scale bar, 20 μm. **b** Hippocampal neurons expressing HDAC6 shRNA. Scale bar, 50 μm. **c** Sholl graphs of neurons expressing Venus or HDAC6 shRNA or HDAC6 shRNA together with shRNA resistant HDAC6. Data represents average of three independent experiments (*n* = 10 for each group in each experiment). **d**-**f** Quantification of the number of primary dendrites (**d**), longest dendrite length (**e**) and total dendrite length (**f**). **g**, **h** HDAC6 did not affect axon specification. The number of axons in neuron was calculated by AnkG positive neurite. **g** Hippocampal neurons expressing Venus (green) or both Venus and HDAC6 shRNA were stained by AnkG (red). Nucleus was stained by Hoechst (blue). Scale bar, 50 μm. **h** Quantification of the percentage of neurons with 0, 1, 2, or more axons. (control, *n* = 62; HDAC6 KD, *n* = 64) *p < 0.05, **p < 0.01 by two-tailed Student’s t-test (unless otherwise indicated). The symbol # represents the number. Error bar means ± S.E.M.

To understand the role of HDAC6 in neuronal development, we downregulated HDAC6 expression in hippocampal neurons using HDAC6 shRNA. Immunocytochemistry experiments of hippocampal neurons showed that the expression of HDAC6 shRNA reduced the HDAC6 level to 44%, but expression of shRNA resistant HDAC6 cDNA in HDAC6 knockdown neurons restored the HDAC6 level to a level similar to the control (Fig. 1a). We found that HDAC6 knockdown neurons transfected at 0 days in vitro (DIV0) had a low number of dendrite crossings at all measured distances as well as a decrease in both the longest and total dendrite length, compared with control cells (Fig. 1b-f). However, the number of primary dendrites was higher in HDAC6 knockdown neurons than in control cells (Fig. 1d). The neuronal defects were rescued by the expression of shRNA resistant HDAC6 cDNA. We also measured the number of axons to examine if HDAC6 influenced axon specification. However, HDAC6 knockdown resulted in no effect on the number of axons (Fig. 1g, h). These results suggest that HDAC6 is required for dendrite development in vitro.

### HDAC6 promotes deployment of the Golgi apparatus into a major dendrite in vitro

**Fig. 2:**
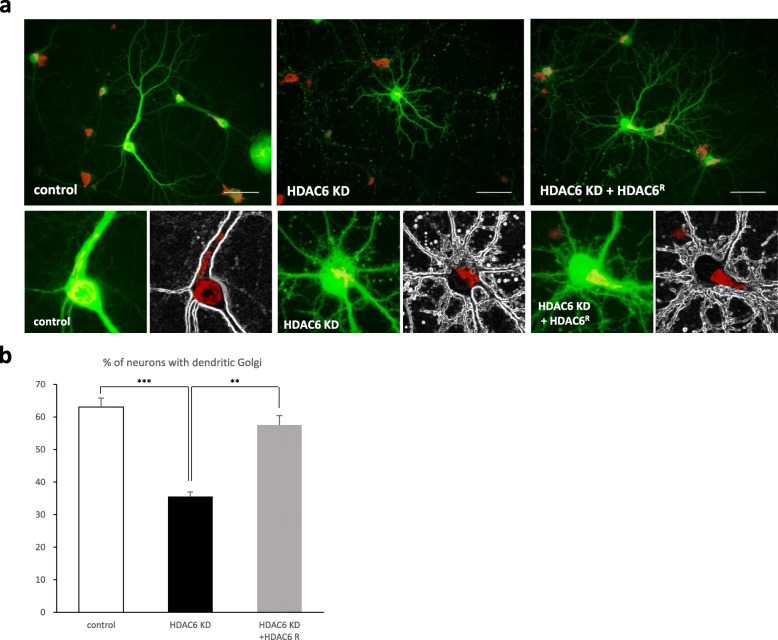
HDAC6 is required for the Golgi polarization into a major dendrite. **a** The Golgi apparatus was stained by GM130 antibody (red). Hippocampal neurons expressing HDAC6 shRNA did not polarize the Golgi into a dendrite. Expression of HDAC6 shRNA together with shRNA resistant HDAC6 polarized the Golgi into a dendrite. Scale bar, 50 μm. **b** Quantification of the percentage of neurons with dendritic Golgi. Data represents average of three independent experiments (*n* = 90–120 for each group in each experiment). **p < 0.01, ***p < 0.001 by two-tailed Student’s t-test. Error bar means ± S.E.M. in all graphs

During neuronal development in vitro, one of primary dendrites becomes the longest and thickest dendrite, called the apical dendrite. In turn, the apical dendrite is where the Golgi apparatus is deployed to support further dendrite development. Because we observed that HDAC6 knockdown neurons did not have thick and long dendrites that are typical of the apical dendrite (Figs. 1b and 2a), we investigated whether the Golgi apparatus was deployed into a dendrite in HDAC6 knockdown neurons. Hippocampal neurons transfected with HDAC6 shRNA at DIV0 were immunostained at DIV14 using anti-GM130 antibody for the localization of the Golgi apparatus. The portion of neurons with the Golgi apparatus polarized into a dendrite was smaller in HDAC6 knockdown neurons than in control cells; the phenotype was rescued by the expression of shRNA resistant HDAC6 cDNA (Fig. 2). These data suggest that defective dendrite development in HDAC6 knockdown neurons is attributed to a lack of Golgi polarization into the dendrite.

### HDAC6 controls dendrite growth through cortactin deacetylation in vitro

**Fig. 3:**
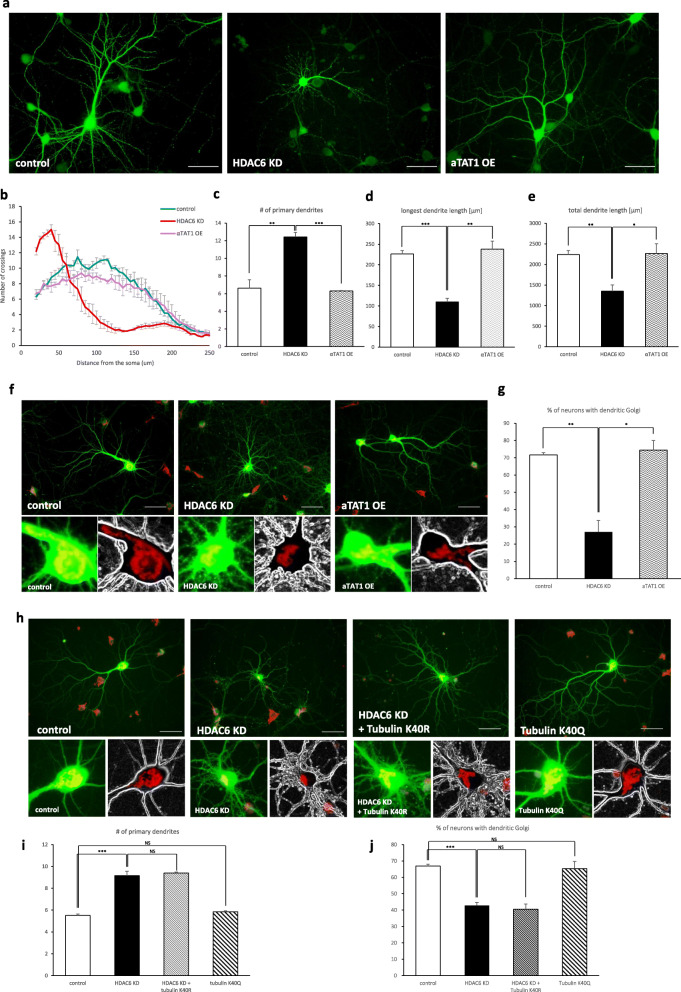
α-tubulin deacetylation by HDAC6 is not related with Golgi polarization. Neurons expressing α-TAT1, α-tubulin acetyl transferase 1, did not change dendrite growth. **a** HDAC6 knockdown neurons affected dendrite growth. αTAT1 overexpressing neurons had normal morphology. Scale bar, 50 μm. **b** Sholl graphs of neurons expressing Venus or HDAC6 shRNA or αTAT1. Data represents average of three independent experiments (*n* = 10 for each group in each experiment). **c**-**e** Graphs represent the number of primary dendrites (**c**), longest dendrite length (**d**), and total dendrite length (**e**). **f** The Golgi apparatus was stained by GM130 antibody (red) in neurons. Unlike HDAC6 knockdown neurons, αTAT1 overexpression neurons did not affect Golgi polarization. Scale bar, 50 μm. **g** Quantification of the percentage of neurons with dendritic Golgi. Data represents average of three independent experiments (*n* = 80–120 for each group in each experiment). **h**-**j** Neurons expressing tubulin acetylation mutants did not influence dendrite development and Golgi polarization. **h** Expression of deacetylation mimetic form of tubulin did not rescue HDAC6 knockdown phenotype. Overexpression of acetylation mimetic form of tubulin did not affect dendrite development and Golgi deployment in primary hippocampal neurons. The Golgi apparatus was stained by GM130 (red). Scale bar, 50 μm. **i**, **j** Quantification of the number of primary dendrites (**i**) and percentage of neurons with dendritic Golgi (**j**). Data represents average of three independent experiments (*n* = 60–120 for each group in each experiment). *p < 0.05, **p < 0.01, ***p < 0.001 by two-tailed Student’s t-test. The symbol # represents the number. Error bar means ± S.E.M. in all graphs

It has been well known that HDAC6 deacetylates α-tubulin and cortactin, which control microtubule dynamics and actin polymerization [19, 20]. To determine whether HDAC6 regulates dendritic growth through α-tubulin, hippocampal neurons were transfected with α-tubulin acetyltransferase 1 (αTAT1), of which overexpression results in hyperacetylation of α-tubulin. However, there were no differences between αTAT1 overexpressing cells and control cells in the number of primary dendrites or in the longest dendrite length, nor in the percentage of neurons with dendritic Golgi (Fig. 3a-g). To further confirm that the acetylation of α-tubulin is not responsible for the defective dendrite development, the deacetyl-mimetic mutant, tubulin K40R, and acetyl-mimetic mutant, tubulin K40Q, were expressed in HDAC6 knockdown and control cells, respectively. No mutant forms showed changes in dendrite morphogenesis or Golgi deployment into a dendrite (Fig. 3h-j).

**Fig. 4:**
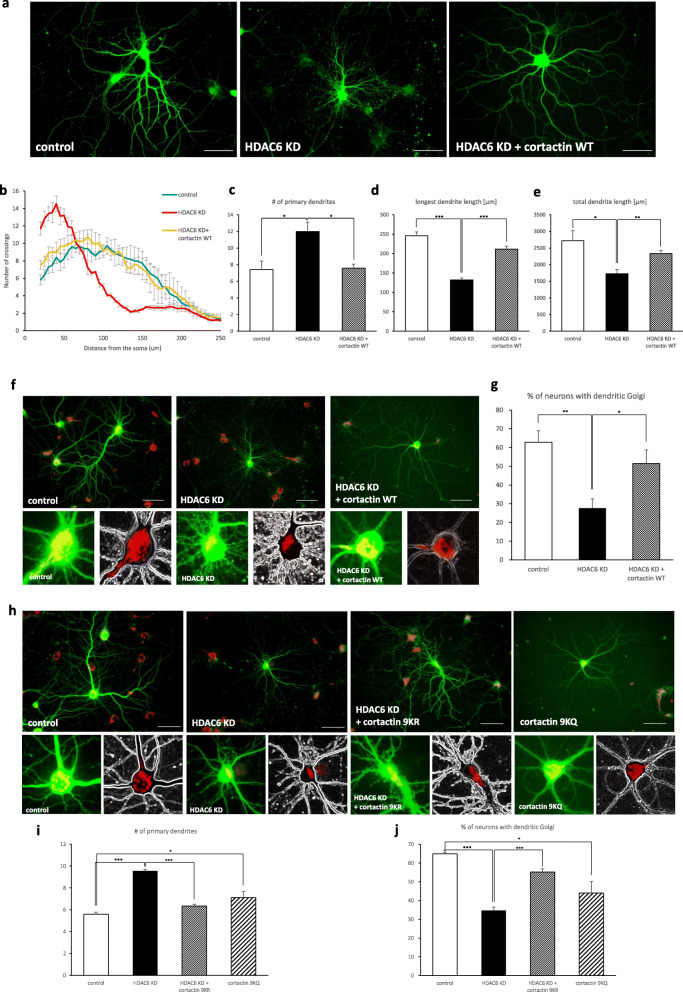
HDAC6 controls dendrite development and Golgi polarization through cortactin deacetylation. Neurons expressing cortactin rescued dendrite growth (**a**-**e**) and Golgi polarization (**f**, **g**) in HDAC6 knockdown neurons. **a** HDAC6 knockdown neurons had abnormal dendrite growth. Neurons expressing HDAC6 shRNA together with cortactin rescued phenotype. Scale bar, 50 μm. **b** Sholl graphs of neurons expressing Venus or HDAC6 shRNA or HDAC6 shRNA together with cortactin. Data represents average of three independent experiments (*n* = 10 for each group in each experiment). **c**-**e** Graphs represent the number of primary dendrites (**c**), longest dendrite length (**d**) and total dendrite length (**e**). **f** The Golgi apparatus was stained by GM130 antibody in neurons. Hippocampal neurons expressing HDAC6 shRNA did not polarize Golgi apparatus. But neurons expressing HDAC6 shRNA with cortactin WT restored Golgi polarization. Scale bar, 50 μm. Acetylation defective or mimetic form of cortactin expression changed dendrite development and Golgi polarization. **g** Quantification of percentage of neurons with dendritic Golgi. Data represents average of three independent experiments (*n* = 80–110 for each group in each experiment). **h**-**j** Acetylation defective or mimetic form of cortactin expression changed dendrite development and Golgi polarization. **h** Expression of deacetylation mimetic form of cortactin rescued HDAC6 knockdown phenotype. Overexpression of acetylation mimetic form of cortactin influenced dendrite development and Golgi deployment in primary hippocampal neurons. The Golgi apparatus was stained by GM130 (red). Scale bar, 50 μm. **i**, **j** Quantification of the number of primary dendrites (**i**) and the percentage of neurons with dendritic Golgi (**j**). Data represents average of three independent experiments (*n* = 40–120 for each group in each experiment). *p < 0.05, **p < 0.01, ***p < 0.001 by two-tailed Student’s t-test. The symbol # represents the number. Error bar means ± S.E.M. in all graphs

To examine whether HDAC6 controls dendritic growth through cortactin deacetylation, after confirming previous results from other researchers that the acetylation level of cortactin increases in HDAC6 knockdown neurons (Additional file 3), we transfected hippocampal neurons with HDAC6 shRNA together with cortactin cDNA. Cortactin expression rescued the HDAC6 knockdown phenotypes, recovering dendritic complexity and the length of longest dendrites (Fig. 4a-e) This suggests that HDAC6 controls dendrite development through cortactin deacetylation. We also determined that cortactin expression restored the ability of HDAC6 knockdown cells to mobilize the Golgi apparatus into a primary dendrite (Fig. 4f, g), indicating that cortactin deacetylation is also involved in Golgi polarization. Additionally, HDAC6 knockdown neurons expressing the deacetyl-mimetic mutant, cortactin 9KR [19], deployed the Golgi apparatus into primary dendrites, whereas expression of the acetyl-mimetic mutant, cortactin 9KQ [19], decreased the number of neurons with dendritic Golgi polarization (Fig. 4h-j). Together, these results indicate that HDAC6 regulates dendrite development through cortactin deacetylation.

### SIRT2 and HDAC6 play a common role in dendrite development

The newly born neurons undergo dramatic morphological changes during neurogenesis and neuronal migration in the cerebral cortex. Major aspects of the morphogenesis of primary neurons in vitro may be recapitulated to acquire polarity in migrating neurons in vivo*,* such as for multipolar-to-bipolar transition and positioning of the Golgi apparatus [38]. Therefore, we sought to determine if HDAC6 plays a role in neuronal migration in the cerebral cortex. For this study, we performed in utero electroporation at embryonic day 14.5 (E 14.5) with plasmids expressing tdTomato (to visualize transfected neurons) and HDAC6 shRNA and analyzed neuronal migration in brain sections of the transfected embryos at E 18.5. However, noticeable change was not observed in the migration of HDAC6 knockdown neurons compared to the control (Additional file 1). This indicates that other deacetylases may be also involved in cortactin deacetylation during cerebral cortex development.

**Fig. 5:**
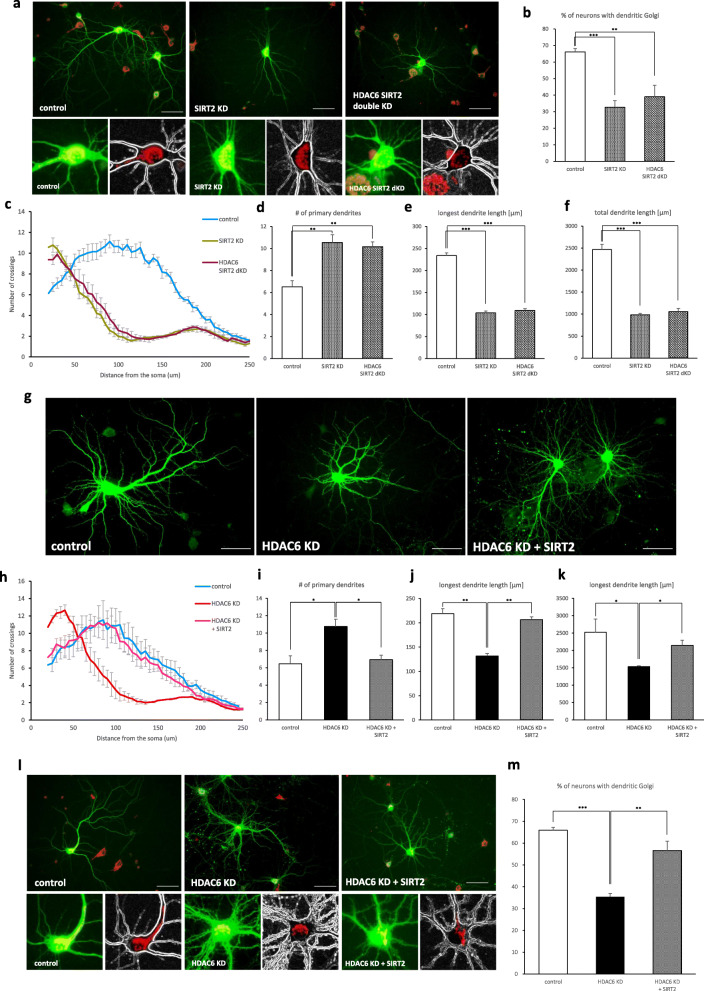
SIRT2 compensates for the function of HDAC6 in hippocampal neurons. **a**-**f** SIRT2 single knockdown or HDAC6 SIRT2 double knockdown was defective in dendrite development and Golgi polarization. **a** Phenotype of SIRT2 knockdown or HDAC6 and SIRT2 double knockdown neurons. The Golgi apparatus was stained by GM130 (red). Scale bar, 50 μm. **b** Graphs showing the percentage of neurons with dendritic Golgi. Data represents average of three independent experiments (*n* = 60–80 for each group in each experiment). **c** Sholl graphs represent the number of crossing in SIRT2 knockdown neurons and HDAC6 and SIRT2 double knockdown neurons. Data represents average of three independent experiments (*n* = 10 for each group in each experiment). **d**-**f** Quantification of the primary dendrites (**d**), longest dendrite length (**e**), and total dendrite length (**f**). **g**-**m** HDAC6 knockdown neurons rescued the phenotype when expressing SIRT2 cDNA. **g** HDAC6 shRNA expressing neurons affected dendrite development. Expression of SIRT2 rescued the HDAC6 knockdown phenotype of hippocampal neurons. Scale bar, 50 μm. **h** Sholl graphs of neurons expressing Venus or HDAC6 shRNA or HDAC6 shRNA together with SIRT2. Data represents average of three independent experiments (*n* = 10 for each group in each experiment). **i**-**k** Quantification of the number of primary dendrites (**i**), longest dendrite length (**j**), and total dendrite length (**k**). **l** Expression of SIRT2 rescued the HDAC6 knockdown phenotype of hippocampal neurons in Golgi polarization. The Golgi apparatus was stained by GM130 (red). Scale bar, 50 μm. **m** Quantification of the percentage of neurons with dendritic Golgi. Data represents average of three independent experiments (*n* = 40–60 for each group in each experiment). *p < 0.05, **p < 0.01, ***p < 0.001 by two-tailed Student’s t-test. The symbol # represents the number. Error bar means ± S.E.M. in all graphs

It has previously been reported that SIRT1, a NAD^ +^ -dependent protein deacetylase, deacetylates cortactin to promote cell migration and increases nerve growth factor-induced neurite outgrowth in PC12 cells [39, 40]. In this study, we demonstrated possible involvement of SIRT1 in neuronal development and migration in the cerebral cortex. Before performing in vivo experiments, the in vitro functions of SIRT1 for dendrite development were assessed. We found that SIRT1 knockdown decreased dendrite complexity but had no effect on dendritic Golgi polarization (Additional file 2). These results are not consistent with the phenotypes of HDAC6 knockdown neurons. Because SIRT2 has also been suggested to be involved in cortactin deacetylation in A549 cells and work synergistically with HDAC6 to promote cell migration and invasion in bladder cancer [19, 41], we examined the role of SIRT2 in dendrite development in vitro. Interestingly, SIRT2 knockdown resulted in a decrease in both dendrite complexity and dendritic Golgi polarization. Simultaneous knockdown of HDAC6 and SIRT2 did not show any additive effect on the defectiveness in dendrite development and Golgi polarization (Fig. 5a-f). This suggests that HDAC6 and SIRT2 may share a common substrate in the dendrite development process. To further clarify that HDAC6 and SIRT2 play a common role in dendrite development, we expressed SIRT2 in HDAC6 knockdown neurons. SIRT2 expression restored the dendritic complexity and dendritic length of HDAC6 knockdown neurons, which was similar to levels found in the control cells (Fig. 5g-k). The defect in Golgi polarization in HDAC6 knockdown neurons was also rescued by SIRT2 expression (Fig. 5l, m). These results suggest that HDAC6 and SIRT2 have a redundant function in dendrite development, although either loss of HDAC6 or SIRT2 is sufficient to cause a defect in dendrite development in vitro.

### Cortactin deacetylation by HDAC6 and SIRT2 regulates neuronal migration and dendrite morphogenesis during cerebral cortex development

**Fig. 6:**
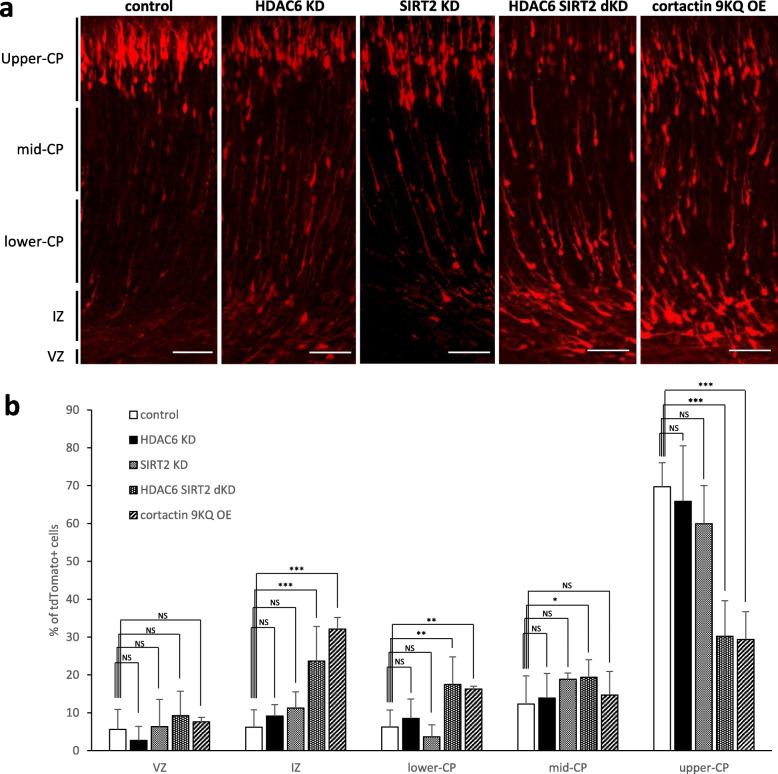
HDAC6 and SIRT2 regulate neuronal migration through cortactin deacetylation in vivo. HDAC6 or SIRT2 single knockdown neurons properly migrated into pial surface. However, HDAC6 and SIRT2 double KD neurons and cortactin acetylation mimetic form expressing neurons did not migrate to the pial surface. **a** HDAC6 and SIRT2 double knockdown neurons did not migrate into cortical plate properly. Neurons overexpressing acetylation mimetic form of cortactin had a defect in radial migration. Scale bar, 50 μm (**b**) Graphs represent the percentage of transfected cells in the cortical layer. *p < 0.05, **p < 0.01, ***p < 0.001 by two-tailed Student’s t-test. Error bar means ± S.D. in all graphs

Because SIRT2 is redundant in function to HDAC6 in dendrite development (Fig. 5), we hypothesized that simultaneous knockdown of HDAC6 and SIRT2 would result in abnormal development of the cerebral cortex. To test the hypothesis, we assessed the effects of HDAC6 and SIRT2 double knockdown on neuronal migration using in utero electroporation of embryonic brains at E 14.5. with plasmids expressing HDAC6 or SIRT2 shRNA. Compared to the control, while HDAC6 or SIRT2 single knockdown neurons did not show any noticeable differences, HDAC6 and SIRT2 double knockdown neurons were significantly slower in neuronal migration (Fig. 6). These results suggest that dysfunction of both HDAC6 and SIRT2 is needed to cause slow neuronal migration in the cerebral cortex. Further, we sought to determine whether the expression of acetyl-mimetic cortactin 9KQ would delay neuronal migration similar to HDAC6 and SIRT2 double knockdown. When cortactin 9KQ was transfected into embryonic brains at E14.5, neuronal migration was retarded in the same manner observed in HDAC6 and SIRT2 double knockdown neurons (Fig. 6). Collectively, these data suggest that cortactin deacetylation by HDAC6 and SIRT2, has a regulatory role in neuronal migration, and lack of either of HDAC6 or SIRT2 is complemented by the other functional one during cerebral cortex development.

**Fig. 7:**
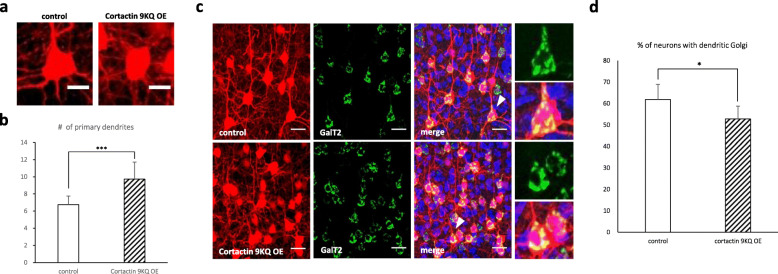
Cortactin deacetylation is important for dendrite growth and Golgi polarization in vivo. Neurons expressing acetylation mimetic form of cortactin were defective in dendrite development and Golgi polarization in vivo*.***a** Phenotype of cortactin acetylation mimetic mutant overexpressing neurons. Scale bar, 20 μm. **b** Quantification of the number of primary dendrites in control and cortactin mutant overexpressing neurons (control, *n* = 32; cortactin 9KQ, *n* = 28). **c**, **d** Acetylation mimetic mutant of cortactin expressing neurons does not polarize dendritic Golgi. Venus-GalT2 was localized in Golgi apparatus. **d** Graph represents the percentage of neurons with dendritic Golgi (control, *n* = 211; cortactin 9KQ, *n* = 188). *p < 0.05, ***p < 0.001 by two-tailed Student’s t-test. The symbol # represents the number. Error bar means ± S.D. in all graphs

Lastly, we sought to ascertain if cortactin deacetylation is essential for dendrite morphogenesis in vivo. To test this, we analyzed dendrite morphology and Golgi polarization in newborn pyramidal neurons at layer II/III in the cerebral cortex. Embryonic brains were transfected using in utero electroporation at E 14.5 with plasmids expressing acetyl-mimetic cortactin 9KQ, together with Venus tagged GalT2 that allowed us to observe Golgi morphology. It was determined that a large portion of neurons expressing cortactin 9KQ in the mouse cerebral cortex failed to form an apical dendrite toward the pial surface; however, they had more primary dendrites (Fig. 7a, b). Furthermore, the percentage of neurons with dendritic Golgi decreased in cortactin 9KQ-expressing cells compared to control neurons (Fig. 7c, d), which is consistent with the in vitro results (Fig. 4h-j). Together, these data suggest that cortactin deacetylation by HDAC6 and SIRT2 is one of major factors regulating neuronal migration and dendrite morphogenesis during cerebral cortex development.

## Discussion

Proper dendrite morphogenesis and multipolar-to-bipolar transition during neuronal migration are crucial for cerebral cortex development and neural circuit formation. Therefore, defects in neuronal morphogenesis during cerebral cortex development ultimately result in aberrant neural connectivity and psychiatric disorders. In this study, to gain a better understanding of the molecular mechanisms underlying the neurogenesis in the brain, we investigated the roles of HDAC6 in the morphogenesis of hippocampal neurons in vitro and the neuronal development during cerebral cortex development. We demonstrated that HDAC6 is necessary for neuronal development. During dendrite arborization of hippocampal neurons, HDAC6 functions to deploy the Golgi apparatus into the apical dendrite. In addition, we demonstrated that SIRT2 has a redundant function with HDAC6 in controlling dendrite morphogenesis and simultaneous knockdown of both HDAC6 and SIRT2 inhibits the neuronal migration during cerebral cortex development through cortactin deacetylation.

α-Tubulin acetylation at lysine 40 residue plays an important role in regulating microtubule functions. Knockdown of histone deacetylase HDAC6 or overexpression of the acetyl-mimetic α-tubulin K40Q reduced the migratory and morphological defects caused by deficiencies in α-tubulin acetyltransferase MEC17 in the cortex [42]. Maternal administration of the histone deacetylase inhibitor tubacin prevented the neuronal migration defects in embryos [43]. Furthermore, expression of α-tubulin K40A, a dominant-negative form that cannot be acetylated, impaired radial migration and reduced dendrite complexity in the cerebral cortex [44]. These previous studies suggest that regulation of acetylation and deacetylation of α-tubulin would be important to promote radial migration and increase dendrite complexity of neurons in the cortex. In this study, we found that expression of αTAT1 or the acetyl-mimetic α-tubulin K40Q had no effect on dendrite morphogenesis, although cortactin deacetylation by HDAC6 and SIRT2 was essential for the dendritic Golgi polarization required for dendrite morphogenesis and neuronal migration in the cortex (Fig. 6). We do not know at the moment why regulation of α-tubulin acetylation or expression of α-tubulin K40Q resulted in different effects on dendrite morphogenesis. More studies in the future will explain why such differences have occurred.

The Golgi apparatus, regulates cargo trafficking and functions as the acentrosomal microtubule-organizing center (MTOC) for the nucleation of microtubules, plays an important role in neuronal cell development [45]. During dendrite morphogenesis, the Golgi enters the apical dendrite, extends toward proximal region, and generates Golgi outposts to support the development of the apical dendrite [46–48]. Therefore, failure to polarize the Golgi apparatus during development may lead to structural and functional damage of the neuronal cells. It is known that Rho GTPases, Rho GEFs, and Rho GAPs control the structure and ultimate position of the Golgi apparatus by regulating actin cytoskeleton dynamics: Cdc42/Rac1-dependent signaling and RhoA-ROCK signaling pathways, αPIX/ARHGEF6, ARFGEF2, and ARHGAP10 are required for appropriate translocation of the Golgi apparatus into dendrites to control neuronal morphogenesis [49, 50]. Interestingly, HDAC6 has been reported to control Golgi fragmentation and cohesion through tubulin deacetylation in human epithelial cells [51]. In this study, we determined that deacetylation of cortactin, but not tubulin, by the histone deacetylases, HDAC6 and SIRT2, was responsible for the control of dendritic Golgi polarization.

Cortactin is localized in the Golgi as well as at the plasma membrane and interacts with a large GTPase, dynamin-2 (Dyn2), to regulate the actin- and Dyn2-dependent transport of cargo. Recruitment of cortactin and Dyn2 to the Golgi is dependent on actin polymerization activated by Arf1, a Arf family GTPase, which regulates membrane trafficking and organelle structure [52]. Recent studies reported that the active GTP-bound form of Arf1 is functionally required for Golgi tubule formation in HeLa cells [53], and BIG2-ARF1-RhoA-mDia1 signaling controls dendritic Golgi polarization in pyramidal neurons in vitro and in vivo [36]. Furthermore, dynamin was found to be involved in the formation of Golgi outposts when activated by RhoA in the Golgi [49]. Although these studies suggest that cortactin participates in the Golgi polarization process, possibly downstream of Arf1, it has previously been unknown whether the regulation of cortactin activity is directly involved in the process of Golgi polarization. In that sense, this study provides an important evidence that modification of cortactin, specifically the acetylation of lysine residues inside the F-actin binding domain of cortactin, is critical for proper Golgi polarization and dendrite morphogenesis. Because acetylated cortactin loses F-actin-binding activity due to the change in the overall charge of the F-actin binding site in cortactin [19, 54], it is possible that cortactin deacetylated by HDAC6 and SIRT2, contributes to dendrite morphogenesis. This may be accomplished by connecting the actin cytoskeleton with dynamin at the Golgi and some scaffolding protein complexes in the plasma membrane. We expect that future research will investigate the questions of how Arf1 or RhoA and the histone deacetylases coordinately regulate the dynamics of and physical interactions of regulators within the actin cytoskeleton, and how these interactions control the Golgi polarization and dendrite morphogenesis in neurons during cerebral cortex development.

## Supplementary information

**Additional file 1.** HDAC6 knockdown alone does not affect neuronal migration in vivo. (PPTX 3222 kb)

**Additional file 2.** SIRT1 is not related with Golgi polarization. (PPTX 1701 kb)

**Additional file 3.** HDAC6 knockdown increases the acetylation status of cortactin. (PPTX 306 kb)

## Data Availability

Not applicable.

## Abbreviations

RGC: Radial glial cell
HDAC6: Histone deacetylase 6
APC: Adenomatous polyposis coli
SIRT1: Sirtuin 1
SIRT2: Sirtuin 2
HSP90: Heat shock protein 90
αTAT1: α-tubulin acetyl transferase 1
Tubulin K40R: tubulin mutant at lysine 40 residue to arginine. The deacetyl-mimetic mutant of tubulin
Tubulin K40Q: tubulin mutant at lysine 40 residue to glutamine. The acetyl-mimetic mutant of tubulin
Cortactin 9KR: cortactin mutant at 9 lysine residues to arginine. The deacetyl-mimetic mutant of cortactin
Cortactin 9KQ: cortactin mutant at 9 lysine residues to glutamine. The acetyl-mimetic mutant of cortactin
DIV: Days in vitro
Dyn2: Dynamin 2
