# Supplementary material for: Cortactin deacetylation by HDAC6 and SIRT2 regulates neuronal migration and dendrite morphogenesis during cerebral cortex development
Source: Mol Brain. 2020 Jul 25;13:105. doi: 10.1186/s13041-020-00644-y (PMC7382832; doi:10.1186/s13041-020-00644-y)
Supplement: Supplementary file 1 — Additional file 1. HDAC6 knockdown alone does not affect neuronal migration in vivo. (PPTX 3222 kb) [file 13041_2020_644_MOESM1_ESM.pptx]

## Slide 1
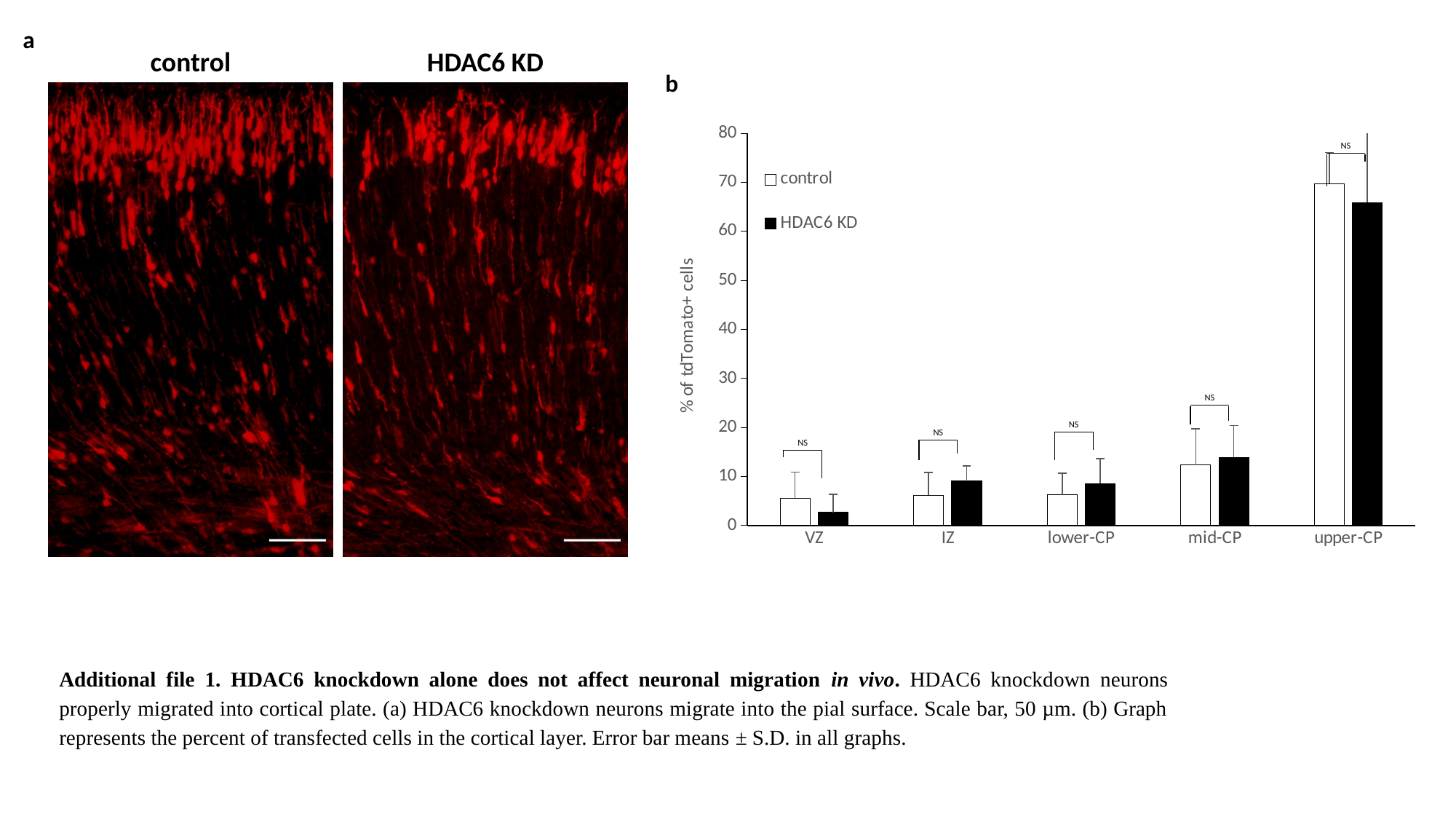

a
control
HDAC6 KD
b
### Chart
| Category | control | HDAC6 KD |
|---|---|---|
| VZ | 5.555758853631194 | 2.6785714285714284 |
| IZ | 6.187923188861862 | 9.091452246940051 |
| lower-CP | 6.227520827802429 | 8.524021709997319 |
| mid-CP | 12.287323946429078 | 13.858438309657823 |
| upper-CP | 69.74147318327543 | 65.84751630483338 |NS
NS
NS
NS
NS
Additional file 1. HDAC6 knockdown alone does not affect neuronal migration in vivo. HDAC6 knockdown neurons properly migrated into cortical plate. (a) HDAC6 knockdown neurons migrate into the pial surface. Scale bar, 50 µm. (b) Graph represents the percent of transfected cells in the cortical layer. Error bar means ± S.D. in all graphs.
