# Supplementary material for: Cortactin deacetylation by HDAC6 and SIRT2 regulates neuronal migration and dendrite morphogenesis during cerebral cortex development
Source: Mol Brain. 2020 Jul 25;13:105. doi: 10.1186/s13041-020-00644-y (PMC7382832; doi:10.1186/s13041-020-00644-y)
Supplement: Supplementary file 2 — Additional file 2. SIRT1 is not related with Golgi polarization. (PPTX 1701 kb) [file 13041_2020_644_MOESM2_ESM.pptx]

## Slide 1
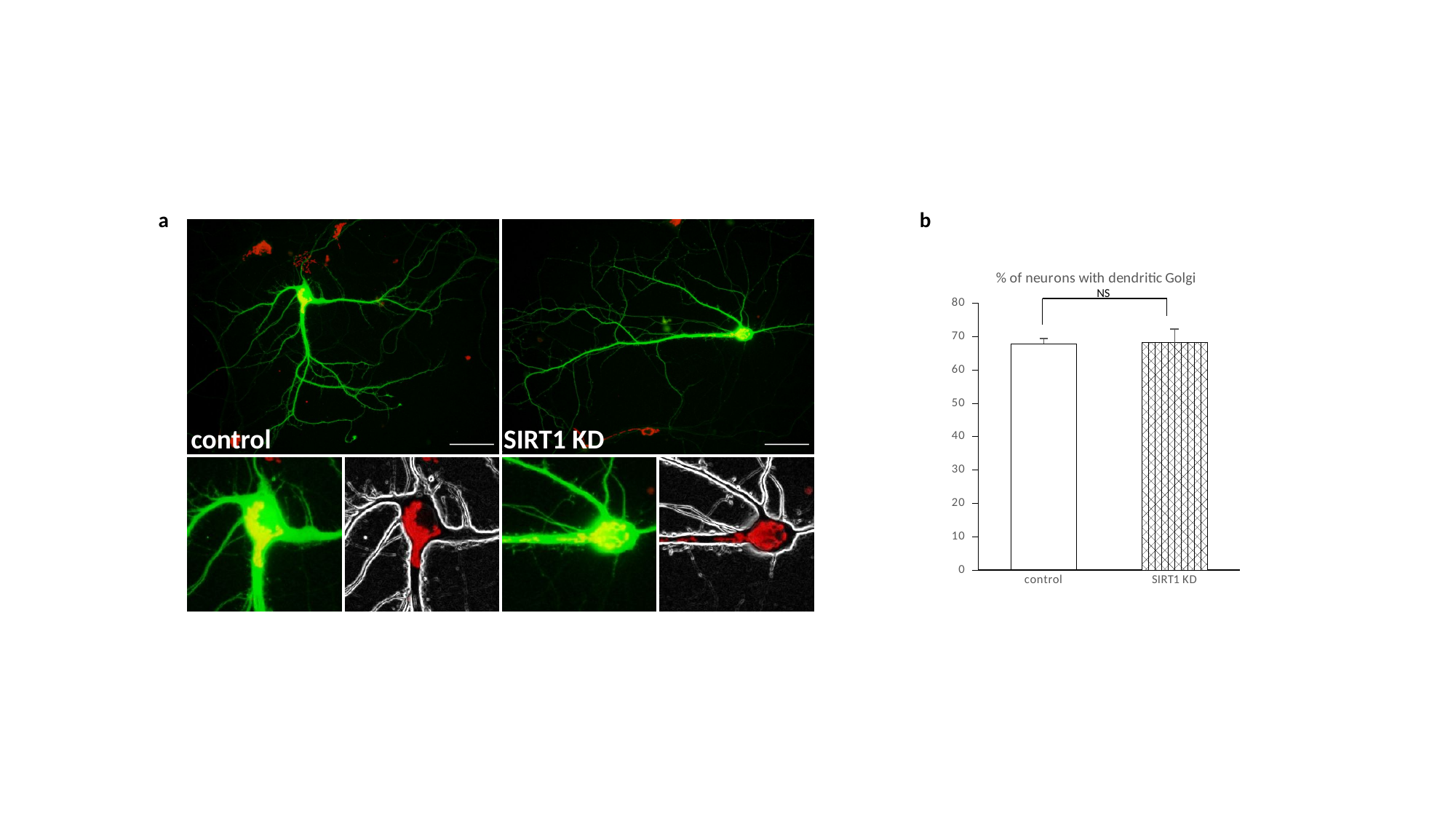

a
b
control
SIRT1 KD
### Chart: % of neurons with dendritic Golgi
| Category | |
|---|---|
| control | 67.91238095238096 |
| SIRT1 KD | 68.22103629083301 |NS

## Slide 2
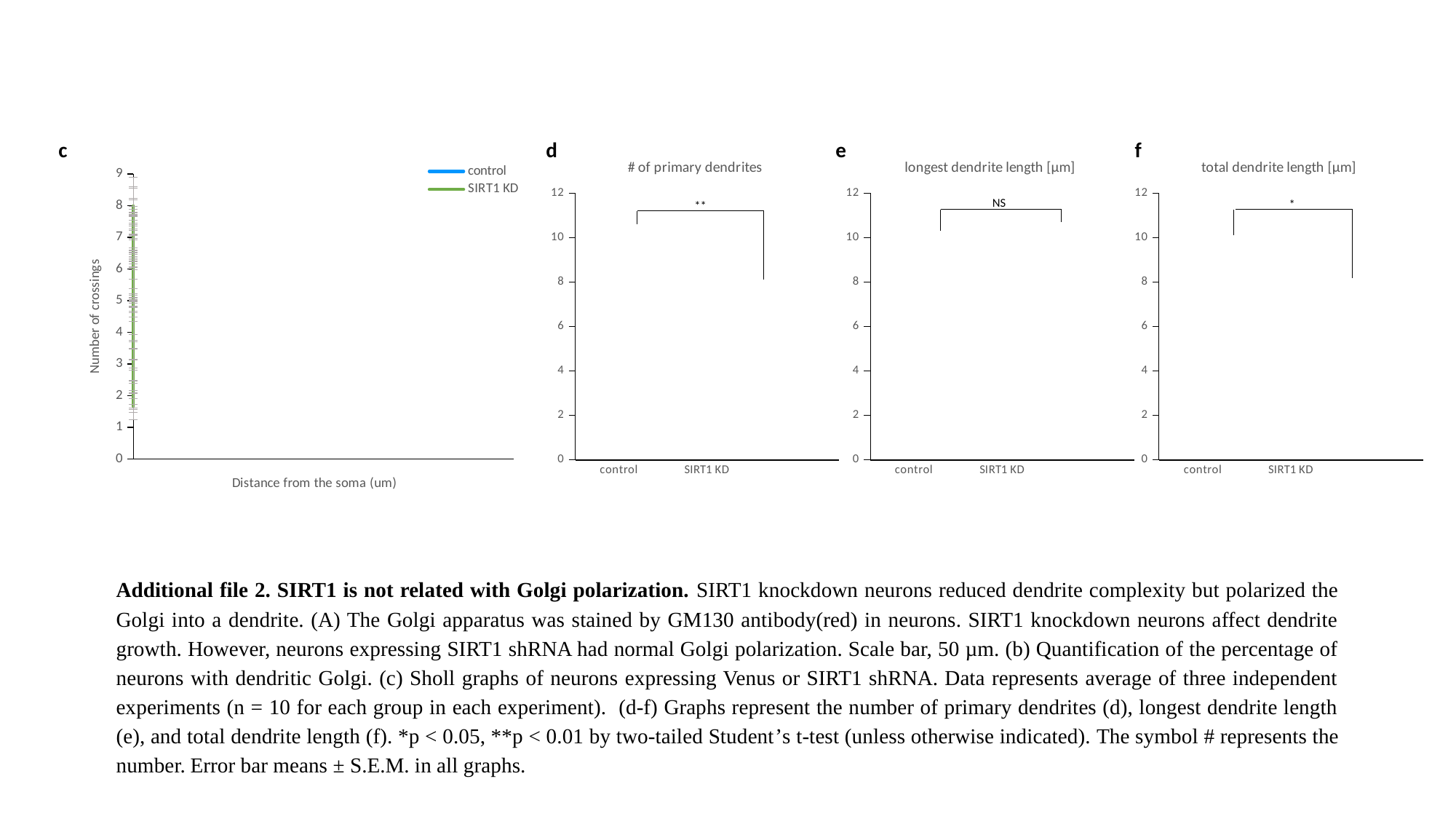

c
d
e
f
### Chart: # of primary dendrites
| Category | |
|---|---|
| control | 5.666666666666667 |
| SIRT1 KD | 4.233333333333333 |
### Chart: longest dendrite length [µm]
| Category | |
|---|---|
| control | 238.56003333333334 |
| SIRT1 KD | 246.31896666666668 |
### Chart: total dendrite length [µm]
| Category | |
|---|---|
| control | 2334.7525 |
| SIRT1 KD | 1747.4124999999997 |
[unsupported chart]
NS
*
**
Additional file 2. SIRT1 is not related with Golgi polarization. SIRT1 knockdown neurons reduced dendrite complexity but polarized the Golgi into a dendrite. (A) The Golgi apparatus was stained by GM130 antibody(red) in neurons. SIRT1 knockdown neurons affect dendrite growth. However, neurons expressing SIRT1 shRNA had normal Golgi polarization. Scale bar, 50 µm. (b) Quantification of the percentage of neurons with dendritic Golgi. (c) Sholl graphs of neurons expressing Venus or SIRT1 shRNA. Data represents average of three independent experiments (n = 10 for each group in each experiment). (d-f) Graphs represent the number of primary dendrites (d), longest dendrite length (e), and total dendrite length (f). *p < 0.05, **p < 0.01 by two-tailed Student’s t-test (unless otherwise indicated). The symbol # represents the number. Error bar means ± S.E.M. in all graphs.
