# Supplementary material for: Cortactin deacetylation by HDAC6 and SIRT2 regulates neuronal migration and dendrite morphogenesis during cerebral cortex development
Source: Mol Brain. 2020 Jul 25;13:105. doi: 10.1186/s13041-020-00644-y (PMC7382832; doi:10.1186/s13041-020-00644-y)
Supplement: Supplementary file 3 — Additional file 3. HDAC6 knockdown increases the acetylation status of cortactin. (PPTX 306 kb) [file 13041_2020_644_MOESM3_ESM.pptx]

## Slide 1
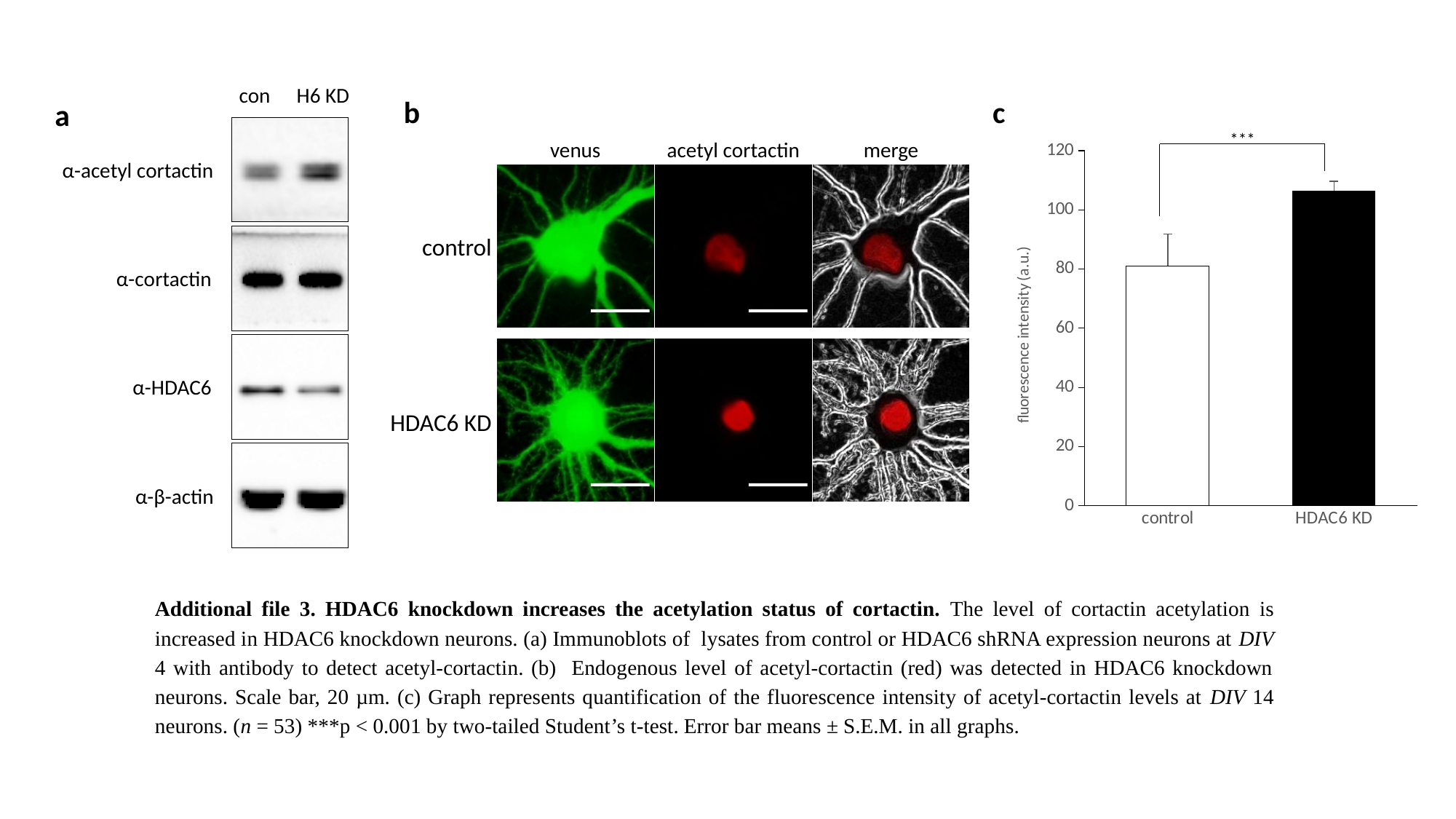

con
H6 KD
b
c
a
***
venus
acetyl cortactin
merge
### Chart
| Category | |
|---|---|
| control | 81.01658823529412 |
| HDAC6 KD | 106.38609041394336 |α-acetyl cortactin
control
α-cortactin
α-HDAC6
HDAC6 KD
α-β-actin
Additional file 3. HDAC6 knockdown increases the acetylation status of cortactin. The level of cortactin acetylation is increased in HDAC6 knockdown neurons. (a) Immunoblots of lysates from control or HDAC6 shRNA expression neurons at DIV 4 with antibody to detect acetyl-cortactin. (b) Endogenous level of acetyl-cortactin (red) was detected in HDAC6 knockdown neurons. Scale bar, 20 µm. (c) Graph represents quantification of the fluorescence intensity of acetyl-cortactin levels at DIV 14 neurons. (n = 53) ***p < 0.001 by two-tailed Student’s t-test. Error bar means ± S.E.M. in all graphs.
